# Genetic liability to inflammatory bowel disease is causally associated with increased risk of erectile dysfunction: Evidence from a bidirectional Mendelian randomization study

**DOI:** 10.3389/fgene.2024.1334972

**Published:** 2024-05-09

**Authors:** Renbing Pan, Chuanyang Sun, Linhai Zheng, Jingwen Liu, Wei Xu

**Affiliations:** ^1^ Department of Urology, The Quzhou Affiliated Hospital of Wenzhou Medical University, Quzhou People’s Hospital, Quzhou, Zhejiang, China; ^2^ Department of Urology, The Second Affiliated Hospital of Soochow University, Suzhou, Jiangsu, China; ^3^ Department of Gastroenterology, The Quzhou Affiliated Hospital of Wenzhou Medical University, Quzhou People’s Hospi, Quzhou, Zhejiang, China; ^4^ Department of Neurology and Psychiatry, Longyou People’s Hospital, Quzhou, Zhejiang, China

**Keywords:** inflammatory bowel disease, erectile dysfunction, genetic association, Mendelian randomization, single nucleotide polymorphisms

## Abstract

**Background:** Several observational cohort studies suggested a close correlation between inflammatory bowel disease and erectile dysfunction. Nevertheless, whether there was a causal effect between them remained debatable. In this study, we aimed to detect the underlying causal links between genetically predicted inflammatory bowel disease and the risk of erectile dysfunction.

**Methods:** A bidirectional Mendelian randomization (MR) study was performed to assess the causal link between inflammatory bowel disease and erectile dysfunction. Inverse variance weighted (IVW), MR-Egger, weighted median, weighted mode, and simple mode were utilized to estimate the causality. The top single nucleotide polymorphisms (SNPs) associated with inflammatory bowel disease cases (*n* = 25,800) and erectile dysfunction cases (*n* = 1,154) were extracted from the summary genome-wide association study (GWAS) data obtained from a publicly attainable database. MR-PRESSO global outlier test and MR-Egger regression were utilized to explore the horizontal pleiotropy and outlier instrumental variables. Cochran’s Q statistic was utilized to detect the heterogeneity.

**Results:** In the forward MR study, the IVW approach demonstrated that genetically determined inflammatory bowel disease exhibited a suggestively causal association with an increased risk of erectile dysfunction (OR: 1.11, 95% CI: 1.02–1.21, *p* = 0.019), and also the genetically determined Crohn’s disease was found to be causally associated with an increased risk of erectile dysfunction (OR: 1.09, 95% CI: 1.02–1.17, *p* = 0.014). However, the MR analysis results showed no significant evidence supporting a causal effect of ulcerative colitis with erectile dysfunction (OR: 1.02, 95% CI: 0.92–1.14, *p* = 0.679). Furthermore, the reverse MR analysis showed no causal effects of genetically determined erectile dysfunction on inflammatory bowel disease. Additionally, sensitivity analysis demonstrated no pleiotropy and heterogeneity.

**Conclusion:** Our MR analysis substantiated causal links of inflammatory bowel disease and Crohn’s disease on erectile dysfunction, which may further elucidate how inflammatory bowel disease impacted the initiation and development of erectile dysfunction, and facilitated the prevention and clinical management of inflammatory bowel disease in individuals with erectile dysfunction.

## 1 Introduction

Inflammatory bowel disease (IBD), as a chronic and recurrent illness, encompasses two primary subtypes, namely, Crohn’s disease (CD) and ulcerative colitis (UC), which are the most prevalent forms (Nishida et al., 2018). Despite UC and CD have various risk factors, including clinical condition, genetic susceptibility, and histologic characteristics, it has been reported that both of them may play a vital impact on endothelial dysfunction in chronic inflammation, furthermore carrying on an elevated risk of cardiovascular disease and sexual dysfunction (Roifman et al., 2009; Wu et al., 2022).

For as we know, the term erectile dysfunction (ED) is defined as the persistent or recurrent incapability to acquire and sustain a sufficiently rigid penile erection statement for satisfactory sexual intercourse (Bacon et al., 2003; Bohm et al., 2007). The condition is prevalent among middle-aged males and elderly individuals (Shiri et al., 2006), with a prevalence rate estimated to range from 20% to 35% (Gleason et al., 2011). The projected global incidence of ED is expected to reach 322 million cases by 2025, as per forecasts (Ayta et al., 1999). The pathophysiology of ED involves the interplay of various factors, including vasculogenic, neurogenic, hormonal, iatrogenic, anatomical, and psychogenic influences (Corona et al., 2006). Indeed, there has been considerable speculation as to whether there exists a correlation between inflammatory bowel disease and an elevated susceptibility to subsequent erectile dysfunction.

So far, there have been some studies exploring the prevalence and correlation between inflammatory bowel disease and ED, but the findings of these studies have exhibited a lack of coherence (Mahmood et al., 2015). Some studies indicated that individuals with inflammatory bowel disease experience significant disruptions in their sexual lives and exhibit a higher prevalence of sexual dysfunction compared to healthy individuals (Mahmood et al., 2015). Furthermore, a previous cohort study conducted by Shmidt et al. revealed that a minimum of 94% of males diagnosed with inflammatory bowel disease experienced erectile dysfunction (Shmidt et al., 2019), and the prevalence of erectile dysfunction prescription was higher among men with inflammatory bowel disease compared to those without the condition (Friedman et al., 2018). However, another cross-sectional observational study involving in 280 male inflammatory bowel disease individuals conducted by Timmer et al. revealed no statistically significant disparity in the International Index of Erectile Function (IIEF) scores between individuals with inflammatory bowel disease and the general individuals, except for sexual lust (Timmer et al., 2007).

To sum up, due to the absence of relevant randomized controlled trial (RCT) studies, the correlation between inflammatory bowel disease and ED is still controversial and obscure. Nevertheless, the association between inflammatory bowel disease and ED in observational cohort studies might be subject to bias due to underlying confounders and reverse causality (Kirkland and Tchkonia, 2017). Thus, it is still indeterminate whether inflammatory bowel disease is the cause or result of ED. Mendelian randomization (MR) analysis, based on genome-wide association studies (GWAS), is a robust approach for constructing instrumental variables (IVs) and inferring causality between two traits in which a causal correlation is difficult to establish utilizing the observational study (Huang et al., 2022). Additionally, MR analysis plays a vital role in separating true causal relationships from links caused by confounders and reverse causal bias (Lawlor et al., 2008). The MR study provided evidence of causality that bridged the gap between traditional epidemiological research and randomized controlled trials (RCTs) (Davies et al., 2018). In this study, we conducted a bidirectional MR method utilizing summary datasets from genome-wide association studies (GWAS) of European ancestry to detect the potential causality between inflammatory bowel disease and ED. Understanding the underlying correlation between inflammatory bowel disease and ED is of great public health importance in complications prevention and disease treatment.

## 2 Materials and methods

### 2.1 Study design

**FIGURE 1:**
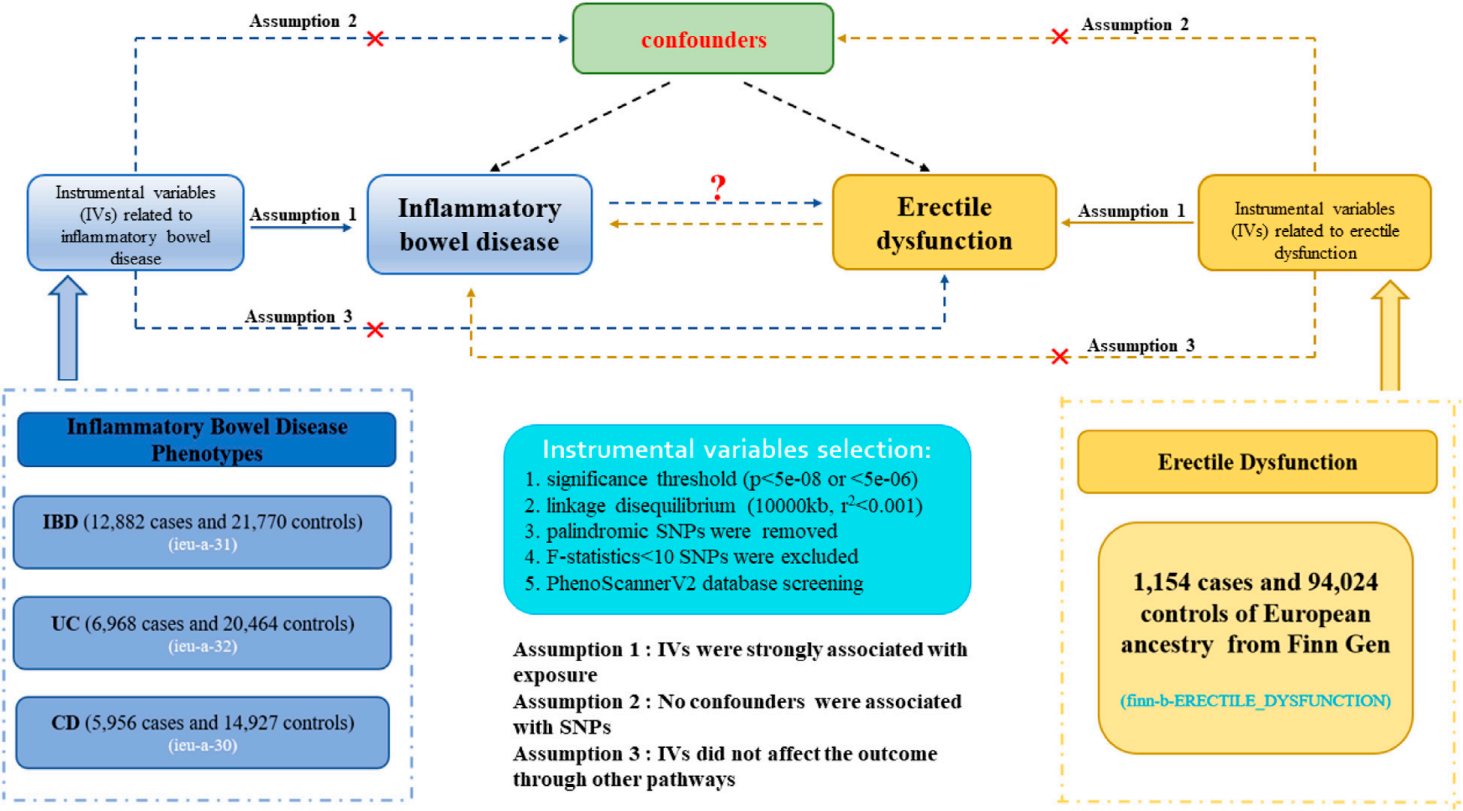
Description of the flow chart in this bidirectional MR study. The blue represented the forward MR analysis, with inflammatory bowel disease as exposure and erectile dysfunction as the outcome. The yellow represented the reverse MR analysis, with erectile dysfunction as exposure and inflammatory bowel disease as the outcome.

Figure 1 demonstrated an explicit flow chart of this bidirectional MR study between inflammatory bowel disease and ED. Our study employed the publicly attainable data from GWAS on inflammatory bowel disease and ED, with detailed information listed in Supplementary Table S1. The forward MR analyses considered inflammatory bowel disease as the exposure and ED as the outcome, and the reverse MR analysis *vice versa*. Relevant single nucleotide polymorphisms (SNPs) were selected from the GWAS summary data through the implementation of quality control procedures. To improve the robustness of the results, this MR study endeavored to fulfill three core assumptions. (1) Instrumental variables (IVs) were strongly associated with the exposure. (2) No confounders were associated with the genetic proxy of exposure (SNPs). (3) IVs affected the outcome only through exposure, and there were no alternative causal pathways for the genetic IVs to impact the outcome. All of the information utilized in this study was derived exclusively from publicly accessible GWAS summary statistics that had been publicly published. Therefore, the study obviated the requirement for ethical approval and individual consent.

### 2.2 Data sources

#### 2.2.1 Inflammatory bowel disease

The GWAS statistics with inflammatory bowel disease were acquired from the international inflammatory bowel disease genetic consortium (IIBDGC) (Liu et al., 2015), which was an international collaboration aiming to facilitate inflammatory bowel disease genetics research. The inflammatory bowel disease datasets (including IBD, UC, and CD) we extracted exclusively consisted of participants of European descent and had the largest sample size. Finally, three inflammatory bowel disease phenotypes were gained from the GWAS datasets, including IBD (N = 12,882 cases and 21,770 controls), UC (N = 6,968 cases and 20,464 controls), and CD (N = 5,950 cases and 14,927 controls). The profile of diverse subtypes data is shown in Supplementary Table S1. All cases and controls were European individuals. In addition, the participants were identified based on radiological, endoscopic, and histopathological assessment.

#### 2.2.2 Erectile dysfunction

The summary datasets for ED individuals were obtained from the publicly attainable GWAS database, including 1,154 cases and 94,024 controls of European ancestry from the Finnish independent cohort (Supplementary Table S1). The analysis was adjusted for principal components and relevant covariates specific to each GWAS, while the overall analysis accounted for principal components. ED cases were identified by doctor-diagnosed, self-reporting, or taking ED drugs. The diagnosed data were then collected through medical records. The absence of overlap occurred due to the acquisition of samples from different consortia for both inflammatory bowel disease and ED cohorts.

### 2.3 Selection of instrumental variables (IVs)

The eligible SNPs were extracted by utilizing various quality control steps. Firstly, the genome-wide significant threshold of *p* < 5e-08 was applied to inflammatory bowel disease and ED to identify robust instrumental variables associated with exposure. When no or few (<3) SNPs were selected for each exposure, we broadened the cutoff to *p*-value < 5e-06 to select eligible IVs. Secondly, to eliminate weak instrument bias, the average of SNPs’ F-statistics was calculated (Burgess et al., 2011; Bowden et al., 2016), and the F-statistics > 10 was regarded as reliable instrumental variables. The F-statistic was a statistical measure that quantifies both the magnitude and accuracy of the genetic impact on the trait. It can be calculated as F = *R* ^2^(N − 2)/(1 − *R* ^2^), where *R* ^2^ represents the proportion of variance in the trait explained by the IVs, and N denotes the sample size of GWAS involving SNPs with the trait (Palmer et al., 2012). Thirdly, the underlying influence of linkage disequilibrium (LD) between single nucleotide polymorphisms (SNPs) was mitigated by implementing an LD threshold for the selected IVs (*r* ^2^ < 0.001, 10,000 kb), assuring the independence of instrumental variables for each exposure. Additionally, the MR-PRESSO global test was used to verify whether the outlier SNPs existed (*p* < 0.05). Fourthly, those IVs associated with the outcome with *p* < 5e-08 (or <5e-06) were removed, and intermediate allele frequency palindromic SNPs were also removed. Lastly, to fulfill the second assumption of MR, SNPs related to confounding factors, encompassing body mass index (BMI), smoking, and blood pressure, were removed by employing the PhenoScannerV2 database (http://www.phenoscanner.medschl.cam.ac.uk/).

### 2.4 Statistical analysis

A variety of approaches, encompassing MR-Egger, weighted median, inverse variance weighted (IVW), simple mode, and weighted mode were utilized to detect the causal associations between inflammatory bowel disease and ED (Burgess and Thompson, 2017). As a meta-analysis method, the random-effects IVW method, which has the best statistical reliability and validity, was regarded as the dominant statistic method to evaluate the underlying bidirectional causal links between inflammatory bowel disease and ED. The IVW method was predicated on the assumption that three fundamental hypotheses hold (Burgess et al., 2013). Considering the correction for multiple comparisons, the Bonferroni correction (*p* = 0.017,0.05/3 exposures) was employed to eliminate the possibility of type I error. This adjustment allowed us to identify a significant causal link among the three different inflammatory bowel disease subtypes. In the forward MR analyses, the exposure variable was assigned to inflammatory bowel disease, while the outcome variable was assigned to ED, and in the reverse MR analyses *vice versa*. Furthermore, other causal analysis methods were also applied as complementary approaches.

### 2.5 Sensitivity analysis

The sensitivity analyses were conducted to evaluate the stability of the results, including MR pleiotropy residual sum and outlier (MR-PRESSO) global test, MR-Egger regression, Cochran’s Q statistic, and leave-one-out (LOO) sensitivity analysis (Dong et al., 2023). Firstly, the MR-PRESSO global test was employed to explore any outliers that could result in pleiotropic biases and to control the effects of pleiotropy by excluding the outliers. Secondly, inspection of horizontal pleiotropy was employed by MR-Egger regression analyses. The significant causal association should also exhibit a non-significant MR-Egger intercept (*p* > 0.05), indicating the absence of horizontal pleiotropy (Chang et al., 2023). Thirdly, Cochran’s Q test was computed to quantify the heterogeneities identified by the IVW and MR-Egger regression approaches. A *p*-value below 0.05 indicated heterogeneity, thereby necessitating the utilization of a random-effect model for subsequent analysis. Additionally, we further investigated whether certain SNPs introduced bias that independently influenced the overall causal estimation, while also assessing the robustness of effect sizes using the LOO sensitivity analysis. All of the MR analysis and sensitivity analysis were carried out in R version 4.2.3, utilizing the R packages “Two Sample MR” and “MR-PRESSO” packages (Hemani et al., 2018).

### 2.6 Candidate mediator selection and two-step MR

To our knowledge, erectile dysfunction was related to common risk factors, encompassing serum folic acid, vitamin D, serum high-sensitivity C-reactive protein, hyperuricemia, and hypertension. Thus, we further performed two-step MR analysis to assess and quantify the mediation effect of each selected mediator in the causal relationship between inflammatory bowel disease diverse subtypes and erectile dysfunction.

## 3 Results

### 3.1 Causal effects of inflammatory bowel disease on ED via forward MR

After palindromic SNP removal, proxy SNP exploration, and the PhenoScannerV2 database filtering, we identified eligible SNPs as IVs to fulfill three core assumptions. The number of SNPs was 62 for IBD, 35 for UC, and 51 for CD. The F-statistic for every SNP was >10. The details and characteristics of IVs for inflammatory bowel disease diverse subtypes were demonstrated in Supplementary Table S2.

**FIGURE 2:**
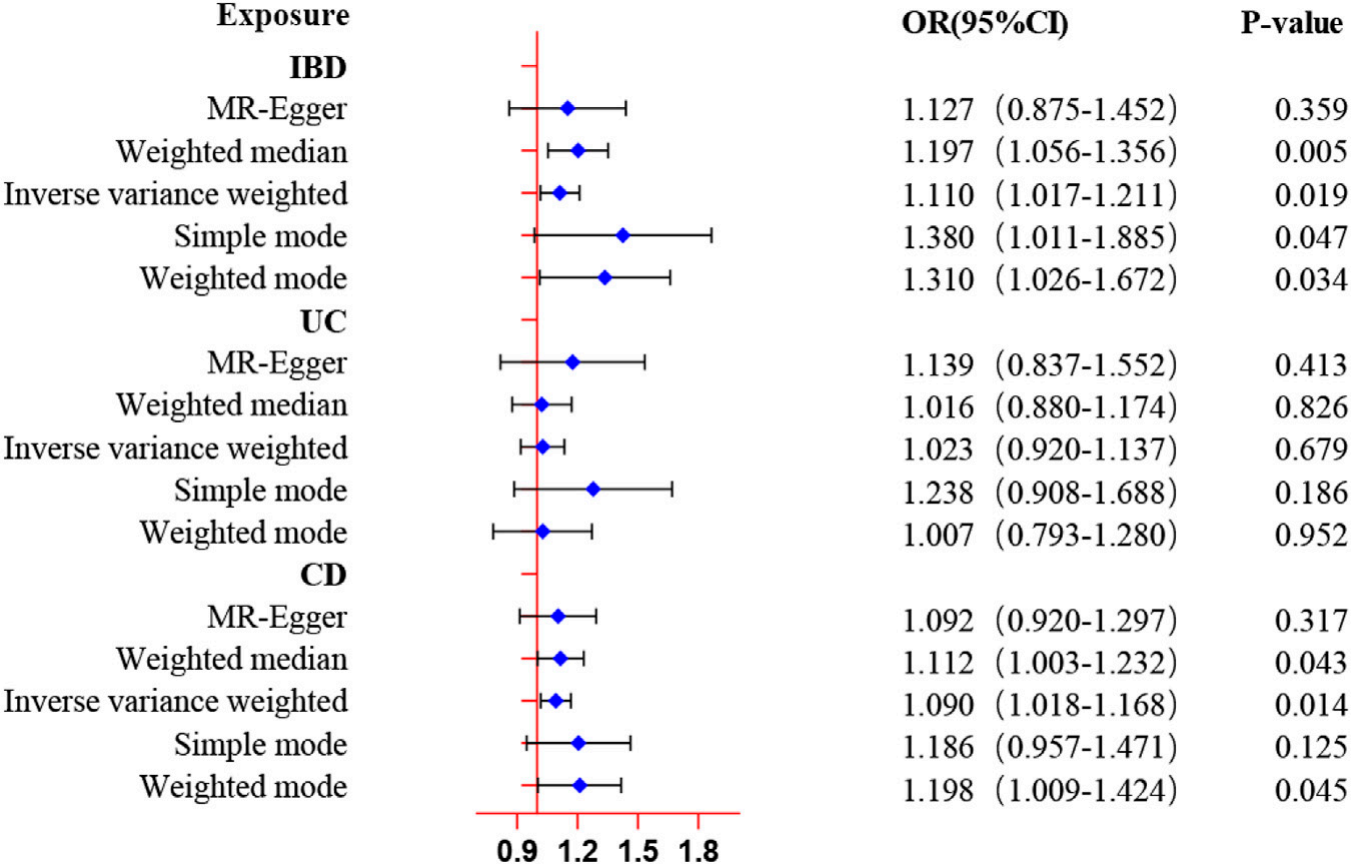
MR estimates of genetically predicted inflammatory bowel disease on ED. The inverse variance weighted method is considered the main method.

The results from the forward MR analysis of the association of inflammatory bowel disease with ED were shown in Figure 2 and Supplementary Table S3. We found the genetically predicted IBD exhibited a suggestively causal association with the increased risk of ED by the IVW approach (OR: 1.11, 95% CI: 1.02–1.21, *p* = 0.019). The findings of the alternative approach demonstrated identical trends and reached statistical significance (OR: 1.20, 95% CI:1.06–1.36, *p* = 0.005 by weighted median; OR: 1.38, 95% CI: 1.01–1.89, *p* = 0.047 by simple mode; OR: 1.31, 95% CI: 1.03–1.67, *p* = 0.034 by weighted mode). Furthermore, the genetically determined CD was also found to be causally associated with an increased risk of ED by the IVW approach (OR: 1.09, 95% CI: 1.02–1.17, *p* = 0.014). Meanwhile, the findings of the weighted median approach and weighted mode method were consistent with the IVW method (OR: 1.11, 95% CI: 1.00–1.23, *p* = 0.043 by weighted median; OR: 1.20, 95% CI: 1.00–1.42, *p* = 0.045 by weighted mode). However, the MR analysis results showed no significant evidence supporting a causal effect of UC with ED (OR: 1.02, 95% CI: 0.92–1.14, *p* = 0.679 by IVW method), and no significant associations were identified in other methods. The scatter plots and LOO sensitivity analysis results of genetically predicted IBD or CD on ED were demonstrated in Supplementary Figure S1. Scatter plots and LOO sensitivity analysis showed that the results were not affected by outliers.

### 3.2 Causal effects of ED on inflammatory bowel disease via reverse MR

Considering the restricted genetic variance, the limited number of IVs and, the low statistical power, we conducted MR analysis by widening the cutoff to *p*-value < 5e-06. Finally, by using this cutoff condition (*r* ^2^ < 0.001, *p* < 5e-06) and excluding the palindromic SNPs, altogether 12 SNPs related to ED were authenticated as IVs in our analysis. The explicit information of the SNPs for ED which were applied in this research manifested in Supplementary Table S4.

**FIGURE 3:**
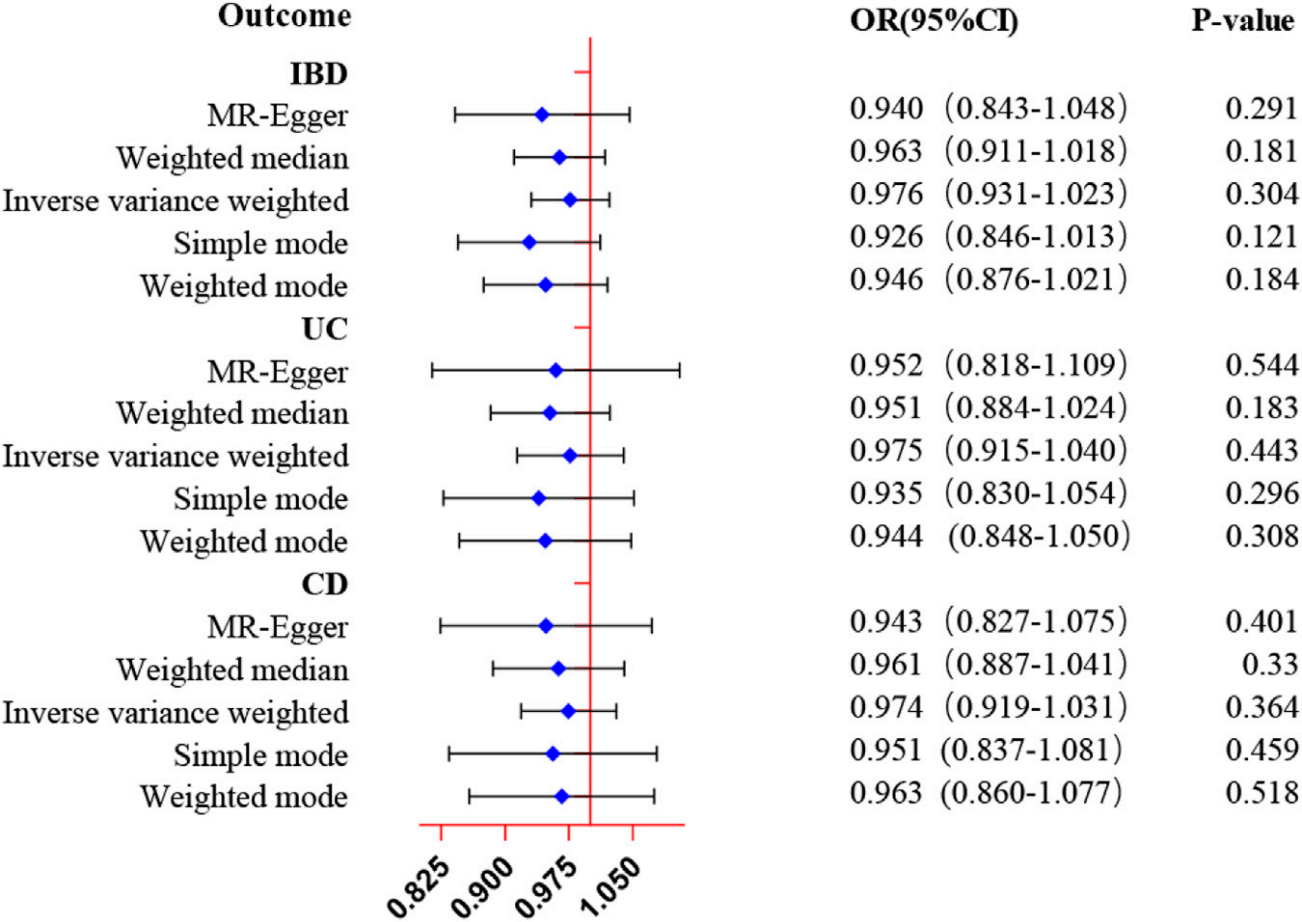
MR estimates of genetically predicted ED on inflammatory bowel disease. The inverse variance weighted method is considered the dominant method.

Reverse MR analysis was conducted to estimate the causal effects of ED on inflammatory bowel disease various subtypes. In contrast to the forward MR analysis, as shown in Figure 3 and Supplementary Table S5, the IVW approach suggested no evidence for the causal effect of genetically determined ED on IBD (OR = 0.98, 95% CI: 0.93–1.02, *p* = 0.304), UC (OR = 0.98, 95% CI: 0.92–1.04, *p* = 0.442), and CD (OR = 0.97, 95% CI: 0.92–1.03, *p* = 0.364). Moreover, the null results were confirmed in the evaluation of other approaches. The scatter plots and forest plots of Mendelian randomization analysis of genetically predicted ED on inflammatory bowel disease were manifested in Supplementary Figures S2 and S3.

### 3.3 Pleiotropy and heterogeneity analysis

For forward and reverse MR analysis, horizontal pleiotropy was not detected in the intercept of MR-Egger regression (Table 1, all *p*-values > 0.05). Additionally, Cochran’s Q statistic indicated significant heterogeneity (Table 1, Q-P_IVW_ = 0.023, Q-P_MR-Egger_ = 0.021) for IVW and MR-Egger method when investigating the MR estimate of UC on ED, so we conducted the IVW approach with random-effect model. The MR-PRESSO global test manifested no horizontal pleiotropy between the IVs and outcomes, and no outlier was identified (Table 1). The sensitivity analysis did not reveal any pleiotropy, and the findings of the LOO sensitivity analysis were not driven by any individual SNPs (Supplementary Figure S4). The funnel plots of MR estimate between genetically predicted inflammatory bowel disease and ED were demonstrated in Supplementary Figure S5.

**TABLE 1:** Heterogeneity and horizontal pleiotropy analysis results.

Exposure	Outcome	Horizontal pleiotropy				Heterogeneity			
		MR-PRESSO global outlier test		MR-Egger regression		MR-Egger		IVW	
		*p-*value	Outlier	Intercept	*p-*value	Q-statistic	*p-*value	Q-statistic	*p-*value
IBD	ED	0.151	no	−0.0025	0.901	72.87	0.123	72.89	0.142
UC	ED	0.030	no	−0.0216	0.469	51.58	0.021	52.42	0.023
CD	ED	0.865	no	−0.0005	0.978	39.09	0.843	39.09	0.867
ED	IBD	0.289	no	0.0119	0.471	12.79	0.236	13.51	0.261
ED	UC	0.172	no	0.0076	0.741	15.82	0.105	16.00	0.141
ED	CD	0.795	no	0.0102	0.606	7.12	0.714	7.40	0.766

### 3.4 Two-step MR estimates for the mediation effect of each mediator in the relationship between IBD and ED

**FIGURE 4:**
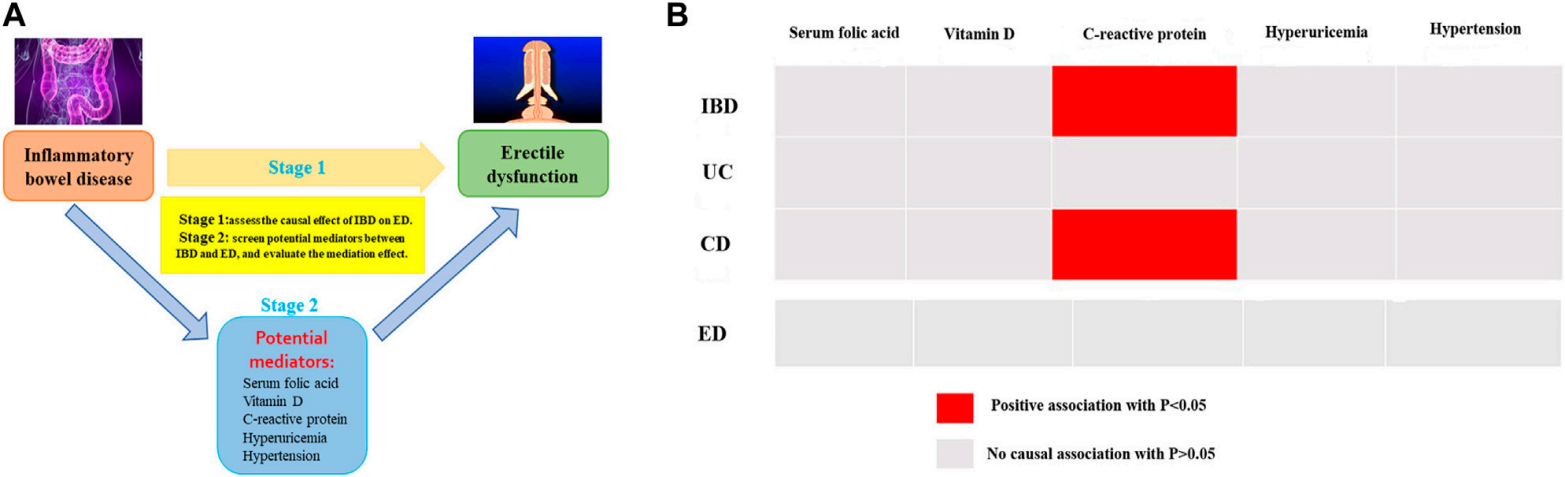
**(A)** The flowchart of two-step MR estimates for the mediation effect of each mediator in the causal association between inflammatory bowel disease and erectile dysfunction **(B)** The results of two-step MR estimates.

Two-step MR analysis was conducted on five potential mediators, including serum folic acid, vitamin D, serum high-sensitivity C-reactive protein, hyperuricemia, and hypertension. The flowchart of the two-step MR analysis was showed in Figure 4A. Nevertheless, by performing two-step MR and quantifying the mediation effect of each selected mediator in the causal link between IBD and ED, no significant association was observed, indicating that the intermediary relationship was not established and these potential factors were not regarded as mediators. The findings of two-step MR estimates were demonstrated in Figure 4B and Supplementary Table S6.

## 4 Discussion

To our knowledge, this is the first MR analysis to explore the causal link between genetically determined inflammatory bowel disease and ED with summary GWAS data. Our two-sample MR study revealed compelling evidence supporting the causal links between genetic predisposition to IBD and CD and the heightened susceptibility to ED in populations of European descent. In contrast, the reverse MR analysis suggested no evidence that genetically determined ED was causally linked to inflammatory bowel disease. In addition, by conducting two-step MR analysis and quantifying the mediation effect of potential factors in the causal relationships between inflammatory bowel disease and erectile dysfunction, no potential mediators were found.

Did inflammatory bowel disease augment the risk of ED? Diverse studies have shown mixed findings (Zhao et al., 2019). Indeed, a previous research performed by Friedman et al. manifested that the relative ratio of ED medication usage was 1.22 among men with inflammatory bowel disease compared to those without the condition. The calculated attributable risk arising from inflammatory bowel disease amounted to 0.022 (Friedman et al., 2018). Furthermore, another study from the Taiwan National Health Insurance Program involving 23 million participants showed that the prevalence of ED was 1.65 times higher in men aged 20 years or older with inflammatory bowel disease compared to those without the condition (Lai et al., 2019). In addition, Kao et al. performed a prospective cohort study to investigate the correlation between inflammatory bowel disease and ED, and they revealed the risk of developing ED in inflammatory bowel disease individuals was 1.64-fold that of the non-inflammatory bowel disease controls (Kao et al., 2016). These studies provided preliminary support for the notion that men afflicted with inflammatory bowel disease faced a significantly heightened susceptibility to experiencing ED. Though these researches have focused on the underlying correlation between inflammatory bowel disease and ED, but no one has revealed the confirmative causal correlation. In the present study, we assessed the causal links between genetic predisposition to inflammatory bowel disease and the risk of ED by performing a two-sample MR study. Coincidentally, we observed that genetic liability to IBD and CD was causally associated with an increased risk of ED, which aligned with previous study results. The results of our research through MR analyses were substantiated by the evidence obtained from the observational study. Unexpectedly, we have not discovered any evidence suggesting causal links between genetically predicted UC and an elevated risk of ED. The variations could be ascribed to the diverse selection of instrumental variables and GWAS datasets, and the inevitable confounding factors. Given the incongruity of the findings, more research was essential to investigate the underlying genetic correlation between UC and ED.

A meta-analysis conducted by X. Wu et al. provided compelling evidence regarding the high prevalence and risk factors of ED in individuals with inflammatory bowel disease (Wu et al., 2022). However, an epidemiological and retrospective study conducted by Marin et al. suggested that male patients with inflammatory bowel disease exhibited significantly poorer outcomes, whereas a higher prevalence of sexual dysfunction was exclusively observed among women (Marin et al., 2013). More intriguingly, the findings from another cross-sectional study conducted by Timmer et al. reported that there was no significant difference in IIEF scores between inflammatory bowel disease patients and the control participants (Timmer et al., 2007), thereby indicating that there was no causal links between inflammatory bowel disease and ED. Nevertheless, in contrast to our MR analysis findings, there was inconsistent and contradictory each other. Actually, the distinction between the impact of chronic inflammatory disease activity itself and the treatment regimen, which included immunosuppressive drugs and biological agents, on a patient’s risk of ED was challenging to ascertain. For instance, a previous study conducted by E. Demir et al. reported that a significant proportion of renal transplant recipients, up to 50%, experienced ED as a result of immunosuppressant therapies, including cyclosporine, low-dose prednisolone, and mycophenolate mofetil (Demir et al., 2006). This adverse effect can have a profound impact on the quality of life for both the patient and his partner (Demir et al., 2006). Moreover, another research conducted by B. Barrou et al. revealed that the selection of a drug therapy for patients with ED in a renal transplant population should be preceded by a thorough assessment of potential interactions with the ongoing immunosuppressive therapy and any potential impact on the graft function (Barrou et al., 2003). They found that sildenafil was effective and safe in transplant individuals with ED (Barrou et al., 2003). The primary metabolic pathway for cyclosporine and tacrolimus was mediated by the hepatic and intestinal cytochrome P450 enzyme, CYP3A4. However, it should be noted that sildenafil acted as an inhibitor of CYP3A4, which may potentially impact the pharmacokinetics of these immunosuppressive drugs and biological agents (Christ et al., 2001). These studies were sufficient to illustrate the crucial role of immunosuppressive drugs and biological agents in inflammatory bowel disease individuals with ED. At present, the accuracy cause of ED in inflammatory bowel disease individuals was still controversial and unknown. In fact, there was a complicated correlation between inflammatory bowel disease and ED, not just a simple cause-and-effect relationship. The connection between inflammatory bowel disease and ED may operate via other common pathways, rather than the diseases themselves. Thus, further investigations were essential to elucidate the potential biological mechanism of how the genetic liability to inflammatory bowel disease affected the initiation and development of ED.

For as we know, this represents the inaugural and methodical MR analysis of the correlations between inflammatory bowel disease and ED. There were 62, 35, and 51 strongly associated and independent SNPs identified for IBD, ulcerative colitis, and Crohn’s disease, respectively. MR analysis results demonstrated an 11% increased risk of ED in IBD individuals and a 9% elevated risk of ED in CD individuals, indicating that IBD or CD is a risk factor for ED. Additionally, in this research, the GWASs have been gradually conducted in the field of inflammatory bowel disease and ED, leading to the identification of several genetic variant susceptibility loci for these two diseases. Despite the enormous human power and financial resources, the highest level of evidence in RCT studies was expected to explore the profound associations between inflammatory bowel disease and ED in the future.

The present study exhibited a causal link between genetically predicted inflammatory bowel disease and the risk of ED. Several strengths of this MR study should be considered. Firstly, the sample sizes of both exposure and outcome were derived from the extensive GWAS datasets. In comparison to previous observational researches, our bidirectional MR analysis effectively mitigated underlying bias, encompassing confounding factors and reverse causation. Secondly, it also assessed the causal correlation between three subtypes of inflammatory bowel disease and ED, which offered further insights into the common pathophysiological mechanism of these two diseases. Thirdly, GWAS statistics of the two cohorts primarily relied on individuals of European descent, thereby effectively mitigating the confounding effects of population stratification. Finally, five causal analytic models and rigorous sensitivity analysis were employed to ensure the resilience and dependability of our findings. Additionally, in view of relevant risk factors for erectile dysfunction, encompassing serum folic acid, vitamin D, serum high-sensitivity C-reactive protein, hyperuricemia, and hypertension, we performed mediated MR analysis to augment the robustness and reliability of the causal effects.

However, our research was also subject to several limitations. First, all datasets utilized in this study were exclusively obtained from individuals of European ancestry, restricting the applicability of our results to diverse ethnic populations. Second, although the findings of the five statistical models were robust, potential bias may arise due to a limited number of genetic instruments or underlying sample repetition between exposure and outcome. Third, as a consequence of the limited scope of MR, only the first assumption could be conventionally tested, while the veracity of the remaining assumptions could not be guaranteed. To satisfy the independent assumption, performing a phenome-wide association study of the instrumental variables may be useful. Fourth, though we utilized multiple methods including MR-Egger regression and MR- PRESSO global outlier test to eliminate horizontal pleiotropy, there may still be residual bias. Fifth, Cochran’s Q test indicated significant heterogeneity for IVW and MR-Egger approach when investigating the MR estimate of UC on ED, suggesting the presence of a handful of solitary SNPs may still have contributed to the potential bias observed in the findings. Finally, the field of GWAS held the potential to unveil insights into the genes implicated in exposure or outcome, yet a comprehensive understanding of the pathophysiology necessitated meticulous mechanistic investigations.

## 5 Conclusion

In conclusion, our research using bidirectional MR study revealed causal links between genetic liability to inflammatory bowel disease and the increased risk of ED. Further research with diverse descent and large-scale datasets was encouraged to further elucidate how inflammatory bowel disease affected the initiation and development of ED.

## Data Availability

The original contributions presented in the study are included in the article/Supplementary Material, further inquiries can be directed to the corresponding authors.
