# Supplementary material for: Genetic liability to inflammatory bowel disease is causally associated with increased risk of erectile dysfunction: Evidence from a bidirectional Mendelian randomization study
Source: Front Genet. 2024 May 9;15:1334972. doi: 10.3389/fgene.2024.1334972 (PMC11112016; doi:10.3389/fgene.2024.1334972)

**Figure S1:** Scatter plots of MR analyses of genetically predicted inflammatory bowel disease on ED; (A) IBD, (C) CD. MR leave-one-out sensitivity analysis to detect whether any individual SNP drove the causal estimates of inflammatory bowel disease on ED; (B) IBD, (D) CD.


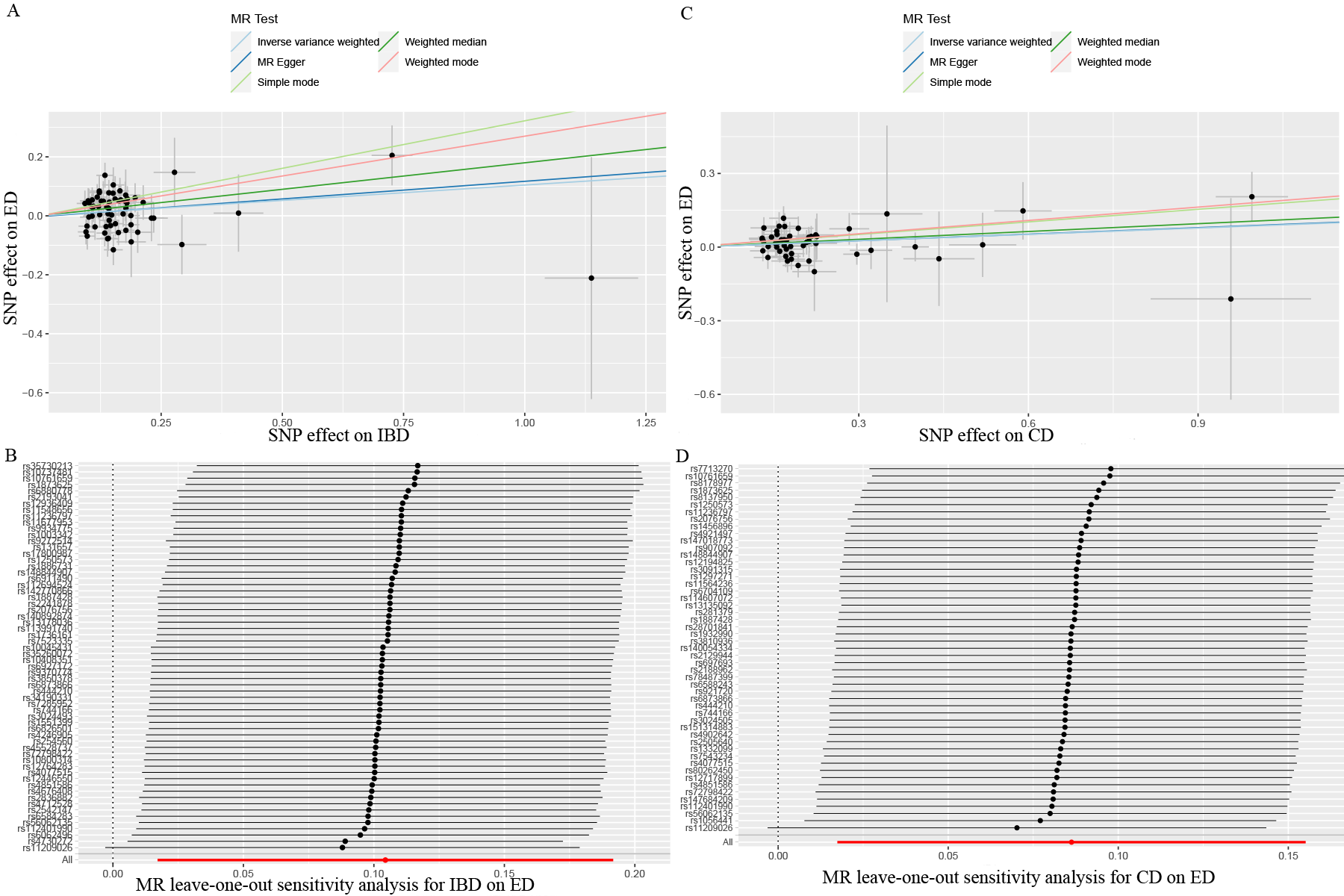

Supplement: Supplementary file 1 [file DataSheet1.ZIP › Supplementary materials/Supplementary Figure S1.docx]
