# Supplementary material for: Genetic liability to inflammatory bowel disease is causally associated with increased risk of erectile dysfunction: Evidence from a bidirectional Mendelian randomization study
Source: Front Genet. 2024 May 9;15:1334972. doi: 10.3389/fgene.2024.1334972 (PMC11112016; doi:10.3389/fgene.2024.1334972)

**Figure S2:** Forest plot of the causal links between inflammatory bowel disease and ED. Legend: (A) MR estimates for IBD on ED. (B) UC on ED. (C) CD on ED. (D) MR estimates for ED on IBD. (E) ED on UC. (F) ED on CD.


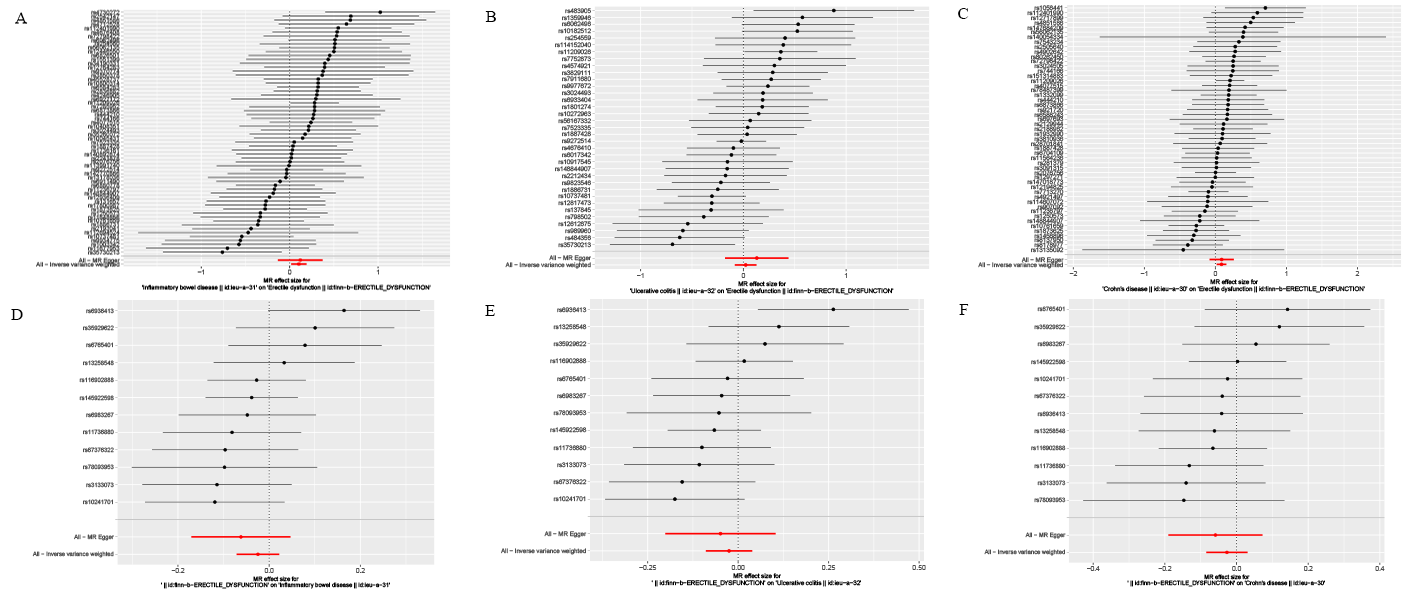


**Figure S3:** Scatter plot of the causal links between inflammatory bowel disease and ED. Legend: (A) MR estimates for IBD on ED. (B) UC on ED (C) CD on ED. (D) MR estimates for ED on IBD. (E) ED on UC. (F) ED on CD.


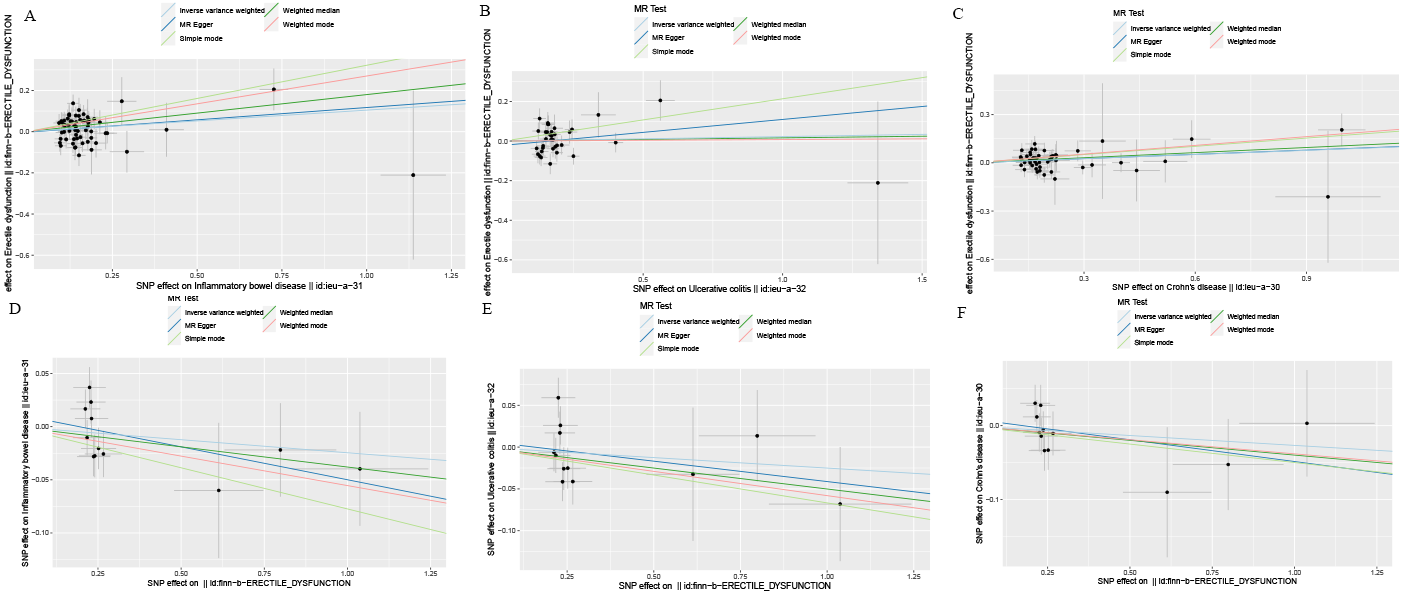


**Figure S4:** Leave-one-out plot of the causal links between inflammatory bowel disease and ED. Legend: (A) MR estimates for IBD on ED. (B) UC on ED. (C) CD on ED. (D) MR estimates for ED on IBD. (E) ED on UC. (F) ED on CD.


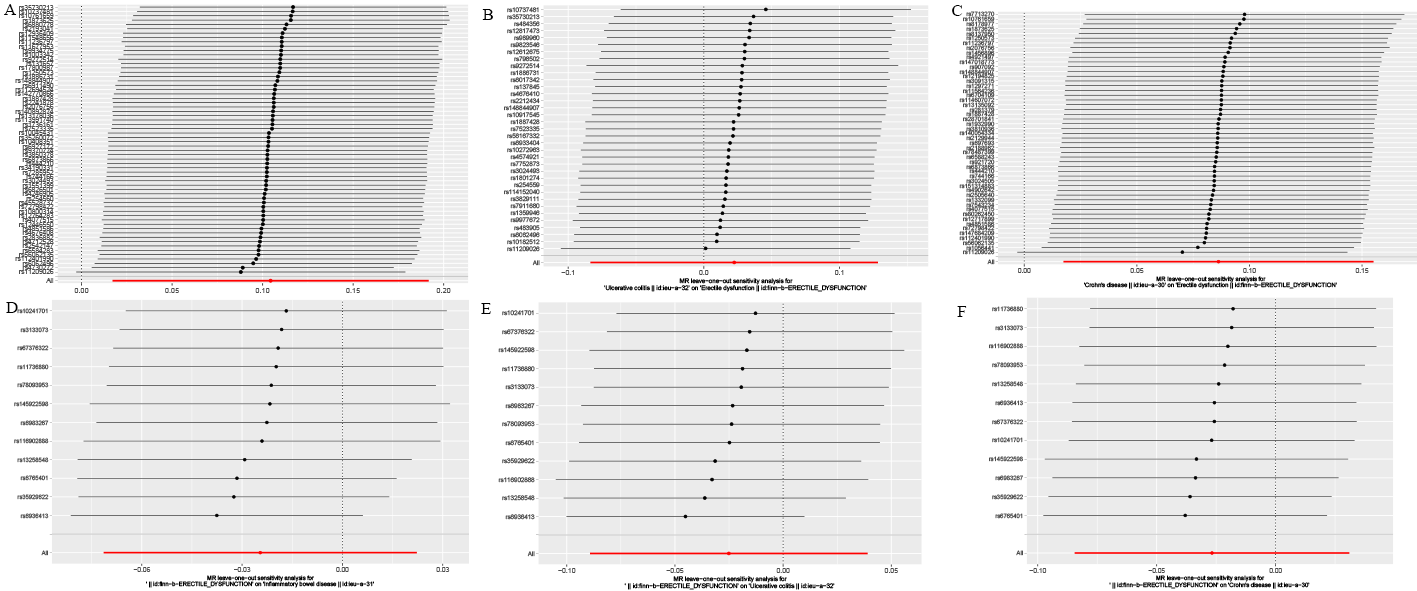


**Figure S5:** Funnel plot of the causal links between inflammatory bowel disease and ED. Legend: (A) MR estimates for IBD on ED. (B) UC on ED. (C) CD on ED. (D) MR estimates for ED on IBD. (E) ED on UC. (F) ED on CD.


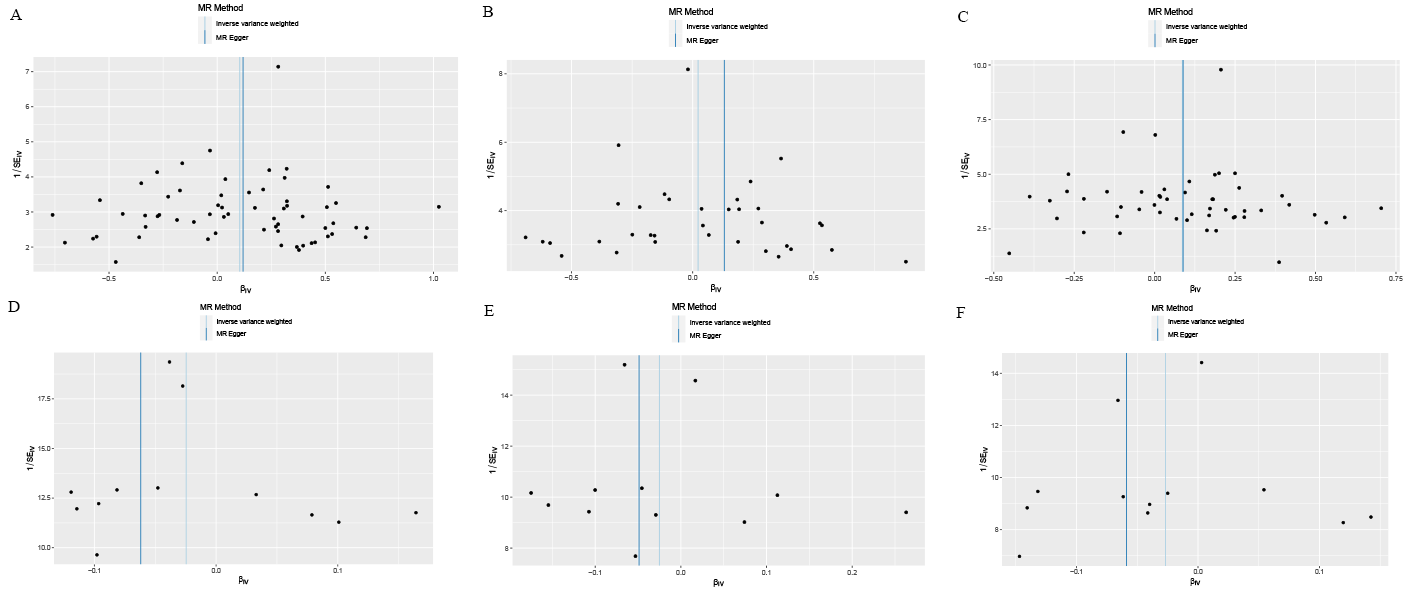

Supplement: Supplementary file 1 [file DataSheet1.ZIP › Supplementary materials/Supplementary Figure S2-S5.docx]
