# Supplementary material for: Genetic liability to inflammatory bowel disease is causally associated with increased risk of erectile dysfunction: Evidence from a bidirectional Mendelian randomization study
Source: Front Genet. 2024 May 9;15:1334972. doi: 10.3389/fgene.2024.1334972 (PMC11112016; doi:10.3389/fgene.2024.1334972)
Supplement: Supplementary file 1 [file DataSheet1.ZIP › Supplementary materials/Supplementary Table S1.docx]

**Table S1**. Brief characteristics description of inflammatory bowel disease and erectile dysfunction GWAS cohorts involved in this study.

| Characteristic | Source | Sample size | Ancestry | Access Link | PMID |
| --- | --- | --- | --- | --- | --- |
| Inflammatory bowel disease (IBD) | IIBDGC | cases:12,882; controls:21,770 | European | <http://gwas.mrcieu.ac.uk/datasets/>  ieu-a-31/ | 26192919 |
| Ulcerative colitis (UC) | IIBDGC | cases:6,968;  controls: 20,464 | European | <http://gwas.mrcieu.ac.uk/datasets/>  ieu-a-32/ | 26192919 |
| Crohn’s disease (CD) | IIBDGC | cases:5,956; controls:14,927 | European | <http://gwas.mrcieu.ac.uk/datasets/>  ieu-a-30/ | 26192919 |
| Erectile dysfunction (ED) | Finn Gen | cases:1,154; controls:94,024 | European | http://gwas.mrcieu.ac.uk/datasets/finn-b-ERECTILE_DYSFUNCTION/ | / |
