# Supplementary material for: Genetic liability to inflammatory bowel disease is causally associated with increased risk of erectile dysfunction: Evidence from a bidirectional Mendelian randomization study
Source: Front Genet. 2024 May 9;15:1334972. doi: 10.3389/fgene.2024.1334972 (PMC11112016; doi:10.3389/fgene.2024.1334972)
Supplement: Supplementary file 1 [file DataSheet1.ZIP › Supplementary materials/Supplementary Table S2.docx]

**Table S2.** Characteristics of instrumental variables used for inflammatory bowel disease in this study (P<5e-08).

| **Inflammatory bowel disease** | **SNP** | **Chr** | **Position** | **Effect allele** | **Beta** | **SE** | **P-value** |
| --- | --- | --- | --- | --- | --- | --- | --- |
| IBD | rs1003342 | 22 | 30570022 | G | -0.0950 | 0.0168 | 1.67×10^-08^ |
| IBD | rs10045431 | 5 | 158814533 | C | 0.1774 | 0.0189 | 6.59×10^-21^ |
| IBD | rs10408351 | 19 | 33754044 | A | 0.1378 | 0.0221 | 4.23×10^-10^ |
| IBD | rs10737481 | 1 | 20171514 | G | 0.1411 | 0.017 | 1.19×10^-16^ |
| IBD | rs10761659 | 10 | 64445564 | G | 0.1619 | 0.0172 | 4.07×10^-21^ |
| IBD | rs10800314 | 1 | 161472789 | A | -0.1431 | 0.0179 | 1.17×10^-15^ |
| IBD | rs11209026 | 1 | 67705958 | A | -0.7263 | 0.0422 | 1.76×10^-66^ |
| IBD | rs11236797 | 11 | 76299649 | A | 0.1557 | 0.017 | 4.75×10^-20^ |
| IBD | rs112401990 | 2 | 61204856 | A | 0.1422 | 0.0174 | 2.84×10^-16^ |
| IBD | rs112694524 | 2 | 43453721 | A | 0.1883 | 0.0303 | 5.39×10^-10^ |
| IBD | rs113991740 | 17 | 32625383 | A | -0.1077 | 0.0189 | 1.32×10^-08^ |
| IBD | rs11548656 | 16 | 81916912 | G | -0.2928 | 0.0507 | 7.72×10^-09^ |
| IBD | rs11677953 | 2 | 219121663 | A | 0.0976 | 0.0171 | 1.05×10^-08^ |
| IBD | rs12446550 | 16 | 28543381 | A | 0.1078 | 0.0171 | 2.78×10^-10^ |
| IBD | rs1250573 | 10 | 81042475 | A | -0.1136 | 0.019 | 2.21×10^-09^ |
| IBD | rs12764283 | 10 | 35530460 | A | 0.1266 | 0.0179 | 1.57×10^-12^ |
| IBD | rs12936409 | 17 | 38043649 | T | 0.1457 | 0.0168 | 3.87×10^-18^ |
| IBD | rs131657 | 22 | 21920817 | A | 0.1365 | 0.0212 | 1.24×10^-10^ |
| IBD | rs13178036 | 5 | 40218529 | C | 0.1009 | 0.0184 | 4.13×10^-08^ |
| IBD | rs140892874 | 12 | 40824798 | C | 0.4096 | 0.0512 | 1.28×10^-15^ |
| IBD | rs142770866 | 19 | 10525372 | A | 0.2300 | 0.0337 | 8.14×10^-12^ |
| IBD | rs148844907 | 6 | 31628397 | A | 1.1375 | 0.0963 | 3.63×10^-32^ |
| IBD | rs1551399 | 8 | 126540200 | C | 0.1013 | 0.0173 | 5.01×10^-09^ |
| IBD | rs1736161 | 21 | 16833222 | A | -0.1233 | 0.0174 | 1.34×10^-12^ |
| IBD | rs17800987 | 5 | 150323428 | G | 0.2017 | 0.0305 | 3.71×10^-11^ |
| IBD | rs1873625 | 3 | 49666964 | A | 0.1773 | 0.0179 | 3.71×10^-23^ |
| IBD | rs1886731 | 1 | 2472081 | C | -0.0971 | 0.0175 | 3.08×10^-08^ |
| IBD | rs1887428 | 9 | 4984530 | C | -0.1716 | 0.0178 | 6.65×10^-22^ |
| IBD | rs2076756 | 16 | 50756881 | G | 0.1876 | 0.0186 | 5.59×10^-24^ |
| IBD | rs2193041 | 12 | 68502110 | G | 0.1337 | 0.0172 | 6.91×10^-15^ |
| IBD | rs2241878 | 2 | 234183718 | C | 0.1480 | 0.0169 | 1.75×10^-18^ |
| IBD | rs2542147 | 18 | 12775851 | T | -0.1513 | 0.0227 | 2.78×10^-11^ |
| IBD | rs254560 | 5 | 134443606 | A | 0.0995 | 0.0171 | 6.16×10^-09^ |
| IBD | rs2836882 | 21 | 40466570 | A | -0.1963 | 0.0201 | 1.49×10^-22^ |
| IBD | rs3024493 | 1 | 206943968 | A | 0.2129 | 0.0222 | 8.48×10^-22^ |
| IBD | rs34190331 | 6 | 111840820 | A | 0.1769 | 0.0303 | 5.39×10^-09^ |
| IBD | rs35260072 | 5 | 131630852 | C | 0.1422 | 0.017 | 7.07×10^-17^ |
| IBD | rs35730213 | 1 | 200874229 | C | -0.1514 | 0.0194 | 6.91×10^-15^ |
| IBD | rs3850378 | 14 | 88452338 | C | 0.1551 | 0.0282 | 3.80×10^-08^ |
| IBD | rs4077515 | 9 | 139266496 | T | 0.1794 | 0.0172 | 1.50×10^-25^ |
| IBD | rs4246905 | 9 | 117553249 | C | 0.1630 | 0.0197 | 1.42×10^-16^ |
| IBD | rs444210 | 6 | 167390242 | G | 0.1095 | 0.0168 | 7.39×10^-11^ |
| IBD | rs45528737 | 4 | 123308465 | T | 0.1668 | 0.03 | 2.66×10^-08^ |
| IBD | rs4676408 | 2 | 241574401 | A | 0.1181 | 0.0181 | 6.62×10^-11^ |
| IBD | rs4712528 | 6 | 20678430 | C | 0.1226 | 0.0207 | 3.07×10^-09^ |
| IBD | rs4730272 | 7 | 107478227 | G | -0.1341 | 0.0178 | 4.50×10^-14^ |
| IBD | rs4851586 | 2 | 103064264 | C | -0.1224 | 0.0193 | 2.32×10^-10^ |
| IBD | rs56062135 | 15 | 67455630 | T | 0.1509 | 0.0198 | 2.64×10^-14^ |
| IBD | rs6062496 | 20 | 62329099 | A | 0.1649 | 0.018 | 5.48×10^-20^ |
| IBD | rs6584283 | 10 | 101290301 | C | -0.1803 | 0.0169 | 1.70×10^-26^ |
| IBD | rs6826501 | 4 | 36076676 | T | -0.0928 | 0.0169 | 4.12×10^-08^ |
| IBD | rs6873866 | 5 | 96249648 | C | -0.1069 | 0.0176 | 1.09×10^-09^ |
| IBD | rs6880778 | 5 | 40399096 | G | 0.1878 | 0.0173 | 2.14×10^-27^ |
| IBD | rs6911490 | 6 | 106522027 | C | -0.1428 | 0.0208 | 6.82×10^-12^ |
| IBD | rs6927172 | 6 | 138002175 | G | 0.1103 | 0.0202 | 4.65×10^-08^ |
| IBD | rs72798422 | 16 | 50866917 | C | 0.2776 | 0.0431 | 1.19×10^-10^ |
| IBD | rs7285952 | 22 | 39733096 | G | -0.1760 | 0.0235 | 7.60×10^-14^ |
| IBD | rs744166 | 17 | 40514201 | G | -0.1207 | 0.0172 | 2.16×10^-12^ |
| IBD | rs7523335 | 1 | 8180210 | A | -0.1405 | 0.0225 | 4.16×10^-10^ |
| IBD | rs9275514 | 6 | 32611650 | T | -0.2347 | 0.0208 | 1.59×10^-29^ |
| IBD | rs9370774 | 6 | 14721897 | C | -0.1307 | 0.0219 | 2.54×10^-09^ |
| IBD | rs9934775 | 16 | 50383077 | T | -0.1396 | 0.0232 | 1.71×10^-09^ |
| UC | rs10182512 | 2 | 61189469 | A | 0.1608 | 0.0223 | 5.19×10^-13^ |
| UC | rs10272963 | 7 | 107486902 | T | -0.1719 | 0.0216 | 1.69×10^-15^ |
| UC | rs10737481 | 1 | 20171514 | G | 0.2501 | 0.0216 | 4.37×10^-31^ |
| UC | rs10917545 | 1 | 20128177 | A | -0.1851 | 0.0335 | 3.29×10^-08^ |
| UC | rs11209026 | 1 | 67705958 | A | -0.5617 | 0.0517 | 1.58×10^-27^ |
| UC | rs114152040 | 5 | 40444986 | A | 0.3396 | 0.0623 | 4.95×10^-08^ |
| UC | rs12612675 | 2 | 219133137 | G | 0.1229 | 0.0219 | 1.98×10^-08^ |
| UC | rs12817473 | 12 | 68497408 | G | 0.1907 | 0.0217 | 1.71×10^-18^ |
| UC | rs1359946 | 13 | 27536972 | A | 0.1583 | 0.0269 | 3.84×10^-09^ |
| UC | rs137845 | 22 | 50439430 | G | 0.1182 | 0.0212 | 2.38×10^-08^ |
| UC | rs148844907 | 6 | 31628397 | A | 1.3413 | 0.1089 | 7.17×10^-35^ |
| UC | rs1801274 | 1 | 161479745 | G | -0.1829 | 0.0217 | 3.78×10^-17^ |
| UC | rs1886731 | 1 | 2472081 | C | -0.1405 | 0.0221 | 2.25×10^-10^ |
| UC | rs1887428 | 9 | 4984530 | C | -0.1767 | 0.0224 | 3.36×10^-15^ |
| UC | rs2212434 | 11 | 76281593 | T | 0.1419 | 0.0213 | 2.46×10^-11^ |
| UC | rs254559 | 5 | 134444982 | A | 0.1243 | 0.0215 | 7.63×10^-09^ |
| UC | rs3024493 | 1 | 206943968 | A | 0.2363 | 0.0276 | 1.09×10^-17^ |
| UC | rs35730213 | 1 | 200874229 | C | -0.167 | 0.0245 | 8.81×10^-12^ |
| UC | rs3829111 | 9 | 139269483 | A | 0.1563 | 0.0214 | 2.89×10^-13^ |
| UC | rs4574921 | 9 | 117538334 | T | 0.1506 | 0.0256 | 4.24×10^-09^ |
| UC | rs4676410 | 2 | 241563739 | A | 0.2078 | 0.0284 | 2.46×10^-13^ |
| UC | rs483905 | 11 | 96023427 | A | 0.1289 | 0.0228 | 1.57×10^-08^ |
| UC | rs484356 | 11 | 114406639 | G | -0.1342 | 0.0228 | 3.95×10^-09^ |
| UC | rs56167332 | 5 | 158827769 | A | 0.1516 | 0.0231 | 5.30×10^-11^ |
| UC | rs6017342 | 20 | 43065028 | C | 0.1913 | 0.024 | 1.38×10^-15^ |
| UC | rs6062496 | 20 | 62329099 | A | 0.1585 | 0.0224 | 1.47×10^-12^ |
| UC | rs6933404 | 6 | 137959235 | C | 0.1668 | 0.0252 | 3.68×10^-11^ |
| UC | rs7523335 | 1 | 8180210 | A | -0.1704 | 0.0285 | 2.29×10^-09^ |
| UC | rs7752873 | 6 | 106579332 | T | 0.1823 | 0.0303 | 1.83×10^-09^ |
| UC | rs7911680 | 10 | 101293468 | C | -0.1718 | 0.0213 | 8.27×10^-16^ |
| UC | rs798502 | 7 | 2789880 | C | -0.1365 | 0.0239 | 1.21×10^-08^ |
| UC | rs9823546 | 3 | 49705512 | A | 0.1769 | 0.0223 | 2.29×10^-15^ |
| UC | rs9891174 | 17 | 38031802 | A | 0.1452 | 0.0212 | 7.17×10^-12^ |
| UC | rs989960 | 7 | 107445727 | T | -0.1291 | 0.0215 | 1.77×10^-09^ |
| UC | rs9977672 | 21 | 40463283 | A | -0.2450 | 0.0261 | 6.21×10^-21^ |
| CD | rs1056441 | 20 | 62370349 | C | 0.167 | 0.0255 | 5.44×10^-11^ |
| CD | rs10761659 | 10 | 64445564 | G | 0.2120 | 0.0237 | 3.42×10^-19^ |
| CD | rs11209026 | 1 | 67705958 | A | -0.9952 | 0.0639 | 1.05×10^-54^ |
| CD | rs11236797 | 11 | 76299649 | A | 0.1811 | 0.0231 | 4.85×10^-15^ |
| CD | rs112401990 | 2 | 61204856 | A | 0.1322 | 0.0237 | 2.35×10^-08^ |
| CD | rs114607072 | 6 | 31351940 | T | 0.4418 | 0.0629 | 2.20×10^-12^ |
| CD | rs11564236 | 12 | 40828306 | T | 0.5191 | 0.0595 | 2.85×10^-18^ |
| CD | rs12194825 | 6 | 20835260 | A | -0.1719 | 0.0298 | 8.00×10^-09^ |
| CD | rs1250573 | 10 | 81042475 | A | -0.1709 | 0.0264 | 9.01×10^-11^ |
| CD | rs12692254 | 2 | 234161211 | T | 0.3014 | 0.0232 | 1.86×10^-38^ |
| CD | rs12717899 | 5 | 141482341 | T | 0.1592 | 0.0289 | 3.59×10^-08^ |
| CD | rs1297271 | 21 | 16823163 | T | -0.1549 | 0.0237 | 6.28×10^-11^ |
| CD | rs13135092 | 4 | 103198082 | G | 0.2215 | 0.0389 | 1.21×10^-08^ |
| CD | rs1332099 | 10 | 101298451 | C | -0.2116 | 0.0231 | 4.36×10^-20^ |
| CD | rs140054334 | 6 | 31587042 | T | 0.3498 | 0.0628 | 2.57×10^-08^ |
| CD | rs1456896 | 7 | 50304461 | T | 0.1393 | 0.0251 | 2.90×10^-08^ |
| CD | rs147018773 | 5 | 150238521 | T | 0.3217 | 0.0375 | 8.89×10^-18^ |
| CD | rs147684209 | 16 | 28870962 | C | 0.1549 | 0.0244 | 2.34×10^-10^ |
| CD | rs148844907 | 6 | 31628397 | A | 0.9580 | 0.1419 | 1.47×10^-11^ |
| CD | rs151314883 | 22 | 39735087 | A | -0.2240 | 0.0327 | 7.12×10^-12^ |
| CD | rs1873625 | 3 | 49666964 | A | 0.1807 | 0.0243 | 1.09×10^-13^ |
| CD | rs1887428 | 9 | 4984530 | C | -0.1681 | 0.0243 | 4.22×10^-12^ |
| CD | rs1932990 | 13 | 44460242 | T | 0.1529 | 0.0263 | 6.02×10^-09^ |
| CD | rs2076756 | 16 | 50756881 | G | 0.3998 | 0.0242 | 3.24×10^-61^ |
| CD | rs2129944 | 19 | 10516198 | G | -0.1562 | 0.0271 | 7.81×10^-09^ |
| CD | rs2188962 | 5 | 131770805 | T | 0.2124 | 0.0228 | 1.36×10^-20^ |
| CD | rs2505640 | 10 | 35459497 | G | -0.1457 | 0.0237 | 7.61×10^-10^ |
| CD | rs281379 | 19 | 49214274 | A | 0.1398 | 0.0238 | 4.26×10^-09^ |
| CD | rs28701841 | 6 | 106530330 | A | 0.2243 | 0.0373 | 1.85×10^-09^ |
| CD | rs3024505 | 1 | 206939904 | A | 0.1779 | 0.0302 | 3.90×10^-09^ |
| CD | rs3091315 | 17 | 32593665 | G | -0.1795 | 0.0263 | 9.52×10^-12^ |
| CD | rs3810936 | 9 | 117552885 | C | 0.2078 | 0.0263 | 2.46×10^-15^ |
| CD | rs4077515 | 9 | 139266496 | T | 0.2159 | 0.0235 | 4.37×10^-20^ |
| CD | rs444210 | 6 | 167390242 | G | 0.1634 | 0.0229 | 1.02×10^-12^ |
| CD | rs4851586 | 2 | 103064264 | C | -0.1689 | 0.0261 | 9.94×10^-11^ |
| CD | rs4902642 | 14 | 69210199 | A | -0.1292 | 0.0236 | 4.34×10^-08^ |
| CD | rs4921497 | 5 | 158848253 | G | 0.1603 | 0.0244 | 5.49×10^-11^ |
| CD | rs56062135 | 15 | 67455630 | T | 0.1931 | 0.0269 | 7.45×10^-13^ |
| CD | rs6588243 | 1 | 67603383 | C | 0.1317 | 0.0234 | 1.78×10^-08^ |
| CD | rs6704109 | 1 | 172857050 | T | 0.2020 | 0.0256 | 2.77×10^-15^ |
| CD | rs6873866 | 5 | 96249648 | C | -0.1681 | 0.0239 | 2.06×10^-12^ |
| CD | rs697693 | 1 | 7886424 | A | 0.1723 | 0.0281 | 8.36×10^-10^ |
| CD | rs72798422 | 16 | 50866917 | C | 0.5904 | 0.0508 | 3.19×10^-31^ |
| CD | rs744166 | 17 | 40514201 | G | -0.1293 | 0.0233 | 2.92×10^-08^ |
| CD | rs7543234 | 1 | 155253308 | T | 0.1555 | 0.0267 | 6.10×10^-09^ |
| CD | rs7713270 | 5 | 40440063 | T | 0.2966 | 0.0241 | 6.97×10^-35^ |
| CD | rs78487399 | 2 | 43809347 | C | 0.2259 | 0.037 | 1.03×10^-09^ |
| CD | rs80262450 | 18 | 12818922 | A | 0.2831 | 0.0353 | 1.08×10^-15^ |
| CD | rs8137950 | 22 | 21969640 | C | 0.1740 | 0.0286 | 1.17×10^-09^ |
| CD | rs8178977 | 19 | 1106477 | C | 0.1928 | 0.0274 | 2.06×10^-12^ |
| CD | rs907092 | 17 | 37922259 | A | 0.1304 | 0.0228 | 1.01×10^-08^ |

Abbreviations: Chr, chromosome, SE, standard error; SNP, single nucleotide polymorphism; IBD, inflammatory bowel disease; UC, ulcerative colitis; CD, Crohn’s disease.
