# Supplementary material for: Genetic liability to inflammatory bowel disease is causally associated with increased risk of erectile dysfunction: Evidence from a bidirectional Mendelian randomization study
Source: Front Genet. 2024 May 9;15:1334972. doi: 10.3389/fgene.2024.1334972 (PMC11112016; doi:10.3389/fgene.2024.1334972)
Supplement: Supplementary file 1 [file DataSheet1.ZIP › Supplementary materials/Supplementary Table S3.docx]

**Table S3.** MR estimate results of inflammatory bowel disease on erectile dysfunction.

| **Exposure** | **Methods** | **nSNP** | **beta** | **SE** | **P-value** | **OR** | **or_lci95** | **or_uci95** |
| --- | --- | --- | --- | --- | --- | --- | --- | --- |
| IBD | MR-Egger | 62 | 0.120 | 0.129 | 0.359 | 1.127 | 0.875 | 1.452 |
|  | Weighted median | 62 | 0.180 | 0.064 | 0.005 | 1.197 | 1.056 | 1.356 |
|  | IVW | 62 | 0.104 | 0.045 | 0.019 | 1.110 | 1.017 | 1.211 |
|  | Simple mode | 62 | 0.322 | 0.159 | 0.047 | 1.380 | 1.011 | 1.885 |
|  | Weighted mode | 62 | 0.270 | 0.125 | 0.034 | 1.310 | 1.026 | 1.672 |
| UC | MR-Egger | 35 | 0.131 | 0.158 | 0.413 | 1.139 | 0.837 | 1.552 |
|  | Weighted median | 35 | 0.016 | 0.073 | 0.826 | 1.016 | 0.880 | 1.174 |
|  | IVW | 35 | 0.022 | 0.054 | 0.679 | 1.023 | 0.920 | 1.137 |
|  | Simple mode | 35 | 0.213 | 0.158 | 0.186 | 1.238 | 0.908 | 1.688 |
|  | Weighted mode | 35 | 0.007 | 0.122 | 0.952 | 1.007 | 0.793 | 1.280 |
| CD | MR-Egger | 51 | 0.088 | 0.087 | 0.317 | 1.092 | 0.920 | 1.297 |
|  | Weighted median | 51 | 0.106 | 0.052 | 0.043 | 1.112 | 1.003 | 1.232 |
|  | IVW | 51 | 0.086 | 0.035 | 0.014 | 1.090 | 1.018 | 1.168 |
|  | Simple mode | 51 | 0.171 | 0.110 | 0.125 | 1.186 | 0.957 | 1.471 |
|  | Weighted mode | 51 | 0.181 | 0.088 | 0.045 | 1.198 | 1.009 | 1.424 |

Abbreviations: SNP: single nucleotide polymorphism; SE: standard error of beta; IVW: Inverse variance weighted; OR: odd ratio.
