# Supplementary material for: Genetic liability to inflammatory bowel disease is causally associated with increased risk of erectile dysfunction: Evidence from a bidirectional Mendelian randomization study
Source: Front Genet. 2024 May 9;15:1334972. doi: 10.3389/fgene.2024.1334972 (PMC11112016; doi:10.3389/fgene.2024.1334972)
Supplement: Supplementary file 1 [file DataSheet1.ZIP › Supplementary materials/Supplementary Table S4.docx]

**Table S4.** Characteristics of instrumental variables used for erectile dysfunction in this study (P<5e-06).

| Exposure | SNP | Chr | Position | Effect allele | Beta | SE | *P*-value |
| --- | --- | --- | --- | --- | --- | --- | --- |
| ED | rs10241701 | 7 | 9237526 | G | 0.2368 | 0.0498 | 2.04×10^-06^ |
| ED | rs116902888 | 18 | 50786387 | T | 0.7984 | 0.1682 | 2.05×10^-06^ |
| ED | rs11736880 | 4 | 159893200 | C | -0.2518 | 0.0527 | 1.76×10^-06^ |
| ED | rs13258548 | 8 | 128496847 | G | -0.2307 | 0.0501 | 4.05×10^-06^ |
| ED | rs145922598 | 17 | 46810586 | T | 1.0376 | 0.2056 | 4.51×10^-07^ |
| ED | rs3133073 | 11 | 134199915 | A | -0.2404 | 0.0475 | 4.26×10^-07^ |
| ED | rs35929622 | 20 | 39611741 | G | -0.2291 | 0.0438 | 1.67×10^-07^ |
| ED | rs67376322 | 4 | 45215338 | A | -0.2664 | 0.056 | 1.95×10^-06^ |
| ED | rs6765401 | 3 | 11917748 | G | 0.2121 | 0.0452 | 2.75×10^-06^ |
| ED | rs6936413 | 6 | 6267630 | G | 0.2247 | 0.0489 | 4.30×10^-06^ |
| ED | rs6983267 | 8 | 128413305 | T | -0.2173 | 0.0424 | 3.09×10^-07^ |
| ED | rs78093953 | 9 | 78947699 | C | 0.6131 | 0.1342 | 4.92×10^-06^ |

Abbreviations: Chr, chromosome; SE, standard error; SNP, single nucleotide polymorphism, ED, erectile dysfunction.
