# Supplementary material for: Genetic liability to inflammatory bowel disease is causally associated with increased risk of erectile dysfunction: Evidence from a bidirectional Mendelian randomization study
Source: Front Genet. 2024 May 9;15:1334972. doi: 10.3389/fgene.2024.1334972 (PMC11112016; doi:10.3389/fgene.2024.1334972)
Supplement: Supplementary file 1 [file DataSheet1.ZIP › Supplementary materials/Supplementary Table S5.docx]

**Table S5.** MR estimate results of erectile dysfunction on inflammatory bowel disease.

| **Outcome** | **Methods** | **nSNP** | **beta** | **SE** | **P-value** | **OR** | **or_lci95** | **or_uci95** |
| --- | --- | --- | --- | --- | --- | --- | --- | --- |
| IBD | MR-Egger | 12 | -0.062 | 0.055 | 0.291 | 0.940 | 0.843 | 1.048 |
|  | Weighted median | 12 | -0.038 | 0.028 | 0.181 | 0.963 | 0.911 | 1.018 |
|  | IVW | 12 | -0.025 | 0.024 | 0.304 | 0.976 | 0.931 | 1.023 |
|  | Simple mode | 12 | -0.077 | 0.046 | 0.121 | 0.926 | 0.846 | 1.013 |
|  | Weighted mode | 12 | -0.055 | 0.039 | 0.184 | 0.946 | 0.876 | 1.021 |
| UC | MR-Egger | 12 | -0.049 | 0.078 | 0.544 | 0.952 | 0.818 | 1.109 |
|  | Weighted median | 12 | -0.050 | 0.038 | 0.183 | 0.951 | 0.884 | 1.024 |
|  | IVW | 12 | -0.025 | 0.033 | 0.443 | 0.975 | 0.915 | 1.040 |
|  | Simple mode | 12 | -0.067 | 0.061 | 0.296 | 0.935 | 0.830 | 1.054 |
|  | Weighted mode | 12 | -0.058 | 0.054 | 0.308 | 0.944 | 0.848 | 1.050 |
| CD | MR-Egger | 12 | -0.059 | 0.067 | 0.401 | 0.943 | 0.827 | 1.075 |
|  | Weighted median | 12 | -0.040 | 0.041 | 0.330 | 0.961 | 0.887 | 1.041 |
|  | IVW | 12 | -0.027 | 0.029 | 0.364 | 0.974 | 0.919 | 1.031 |
|  | Simple mode | 12 | -0.050 | 0.065 | 0.459 | 0.951 | 0.837 | 1.081 |
|  | Weighted mode | 12 | -0.038 | 0.057 | 0.518 | 0.963 | 0.860 | 1.077 |

Abbreviations: SNP: single nucleotide polymorphism; SE: standard error of beta; IVW: Inverse variance weighted; OR: odd ratio.
